# Physiological Adaptations, Nutritional Strategies, and Health Management in Aquatic Organisms

**DOI:** 10.3390/biology15040324

**Published:** 2026-02-12

**Authors:** Quanquan Cao

**Affiliations:** Fisheries College, College of Animal Science and Technology, Sichuan Agricultural University, Chengdu 611130, China; qq2312159@sicau.edu.cn

Aquatic organisms are continuously challenged by dynamic environmental factors, including temperature, salinity fluctuations, and dissolved oxygen levels [1,2,3,4,5,6,7,8]. In this Special Issue, Zhai et al. [9] delve into thermal sensitivity by integrating oxygen consumption rates and transcriptome analysis to reveal the energy metabolism mechanisms in wild Amur grayling (*Thymallus grubii*) under acute warming. Similarly, Wang et al. [10] explore the adaptation process of *Huso dauricus* to high temperatures through the lens of intestinal microbiota succession, while Dao et al. [11] investigate the metabolic adaptation strategies of fish on the Yun-Gui Plateau following temperature acclimation. Beyond thermal stress, aquatic animals must also cope with hypoxic conditions and salinity variations. Yan et al. [12] focus on the acute effects of hypoxia and reoxygenation on physiological indices in hybrid catfish. Furthermore, building on the broader understanding of osmoregulation in aquatic animals [13], Zhang et al. [14] demonstrate the molecular adaptation mechanisms of the hybrid Erythroculter in saline–alkali water, highlighting the potential for aquaculture expansion in non-conventional waters.

Nutritional regulation is pivotal for optimizing growth and bolstering health, particularly through the modulation of intestinal barrier function and immunity by functional nutrients such as amino acids [15,16,17] and vitamins [18]. Addressing the industry’s need for sustainable protein sources—and the associated challenges regarding intestinal integrity [19]—Cao et al. [20] report that substituting fish meal with cottonseed protein induces hepatic ferroptosis in largemouth bass (*Micropterus salmoides*) via the SIRT1-YAP-TRFC axis. To mitigate metabolic disturbances, functional additives play a crucial role. Contributing to this field, Zhao et al. [21] reveal that dietary methionine hydroxy analog regulates hepatic lipid metabolism via SIRT1/AMPK signaling pathways. Additionally, Meng et al. [22] demonstrate the benefits of dietary *Rhodotorula mucilaginosa* on muscle composition in red claw crayfish, and Zhang et al. [23] evaluate the antioxidant-enhancing effects of vitamin K3 in coho salmon (*Oncorhynchus kisutch*) alevins. Notably, Cao et al. [24] underscore the synergistic power of nutrition and environment by examining the beneficial effects of arginine supplementation on yellow catfish specifically under low-temperature farming conditions.

Finally, sustainable aquaculture requires a holistic perspective that spans from genomic insights for disease resistance [25] to macroscopic ecological management. The current issue expands on this scope through three ecological studies. Cai et al. [26] analyze the guild interactions hindering the population recovery of large yellow croakers. Rusco et al. [27] explore the productivity and quality benefits of Integrated Multi-Trophic Aquaculture (IMTA) systems. On a broader biogeographical scale, Nhat et al. [28] reveal the impact of submarine groundwater discharge on the spatial variability of coastal fish diversity using environmental DNA (eDNA) technology.

Taken together, the studies presented in this Special Issue highlight the complex interconnections between biodiversity, physiological adaptation, nutritional regulation, and environmental variability.

## Data Availability

Data available on request from the authors.
